# Broken heart after heart attack- post-MI Takotsubo syndrome: a case report

**DOI:** 10.11604/pamj.2025.51.101.43609

**Published:** 2025-08-21

**Authors:** Nadeem Kassam, Willy Mucyo, Mzee Ngunga, Mohamed Jeilan, Mohamed Varwani

**Affiliations:** 1 Section of Cardiology, Aga Khan University Hospital, Nairobi, Kenya

**Keywords:** Takotsubo cardiomyopathy, ST-segment elevation MI, interrelated, case report

## Abstract

Takotsubo cardiomyopathy (TCM) is a common differential for acute myocardial infarction (AMI), and although its coexistence is considered rare, it may be more common than previously believed. Diagnosing TCM after an AMI is particularly challenging, as initial imaging typically supports AMI. We report the case of a 61-year-old male who presented with anterior ST-elevation myocardial infarction (STEMI) and underwent successful primary percutaneous coronary intervention (PCI) to the left anterior descending artery. Initial echocardiography demonstrated preserved apical function; however, the patient was readmitted with chest pain, diaphoresis, and dyspnea. Suspecting stent thrombosis, repeat coronary angiography confirmed patent stents, but echocardiography revealed severe left ventricular dysfunction with apical ballooning, consistent with TCM. This case underscores the importance of considering TCM as a possible concomitant or subsequent condition in patients with recent Acute Coronary Syndrome (ACS).

## Introduction

Takotsubo cardiomyopathy (TCM) is an acute, reversible condition that clinically mimics an AMI [1]. It is characterized by sudden and regional left ventricular dysfunction in the absence of angiographic evidence of obstructive coronary artery disease [1,2]. Recent reports have suggested that these two discrete conditions coexist [1]. The term Takotsubo, first described in 1990 in Japan, translates into an octopus trap resembling the systolic apical ballooning of the left ventricular (LV) cavity [3]. The incidence of TCM among troponin-positive patients suspected of ACS is less than 2% [2,3]. Registry studies have reported trends to be much more common in women than men and to occur in older adults, with a mean age of 64 years [2,3]. Herein, we report a unique case of TCM with concomitant obstructive coronary artery disease.

## Patient and observation

**Patient information:** a 61-year-old Caucasian male with a background of uncontrolled diabetes mellitus, hypertension, and dyslipidemia presented to our emergency department with a 48-hour history of acute worsening typical chest pain characterized as heaviness and pressure associated with difficulty in breathing, awareness of heartbeat, and diaphoresis.

**Figure 1:**
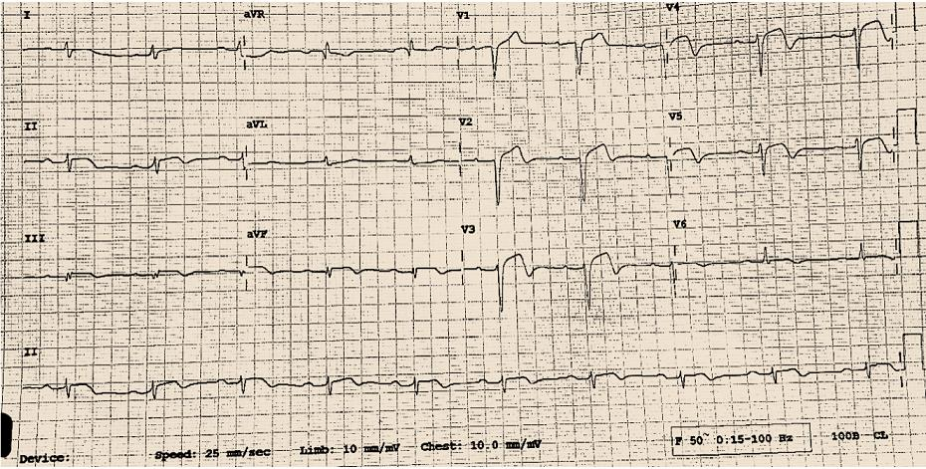
normal sinus rhythm, heart rate of 80bpm, and ST-elevation in the anterior leads as seen

**Clinical findings:** on examination, the patient was found to be restless, afebrile and in severe distress with the following vitals: blood pressure (BP) of 210/100 mmHg, heart rate (HR) of 85 beats/min, and a respiratory rate of 26 cycles/min with saturation of >94% on room air. An initial electrocardiography (ECG) done on admission revealed a sinus rhythm, HR of 80 bpm, and ST-elevation in the anterior leads as seen in Figure 1.

**Diagnostic assessment and therapeutic interventions:** eighty percent stenosis of the left anterior descending artery, at the first diagonal bifurcation (red arrow), and 80.

**Figure 2:**
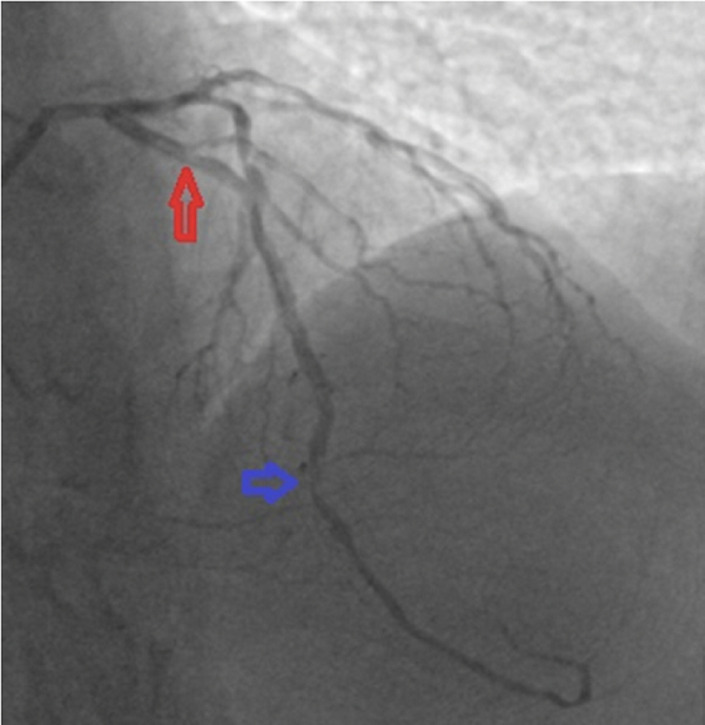
coronary angiogram: postero-anterior cranial view showing 80% stenosis of the left anterior descending artery, at the first diagonal bifurcation (red arrow), and 80% mid stenosis (blue arrow)

**Figure 3:**
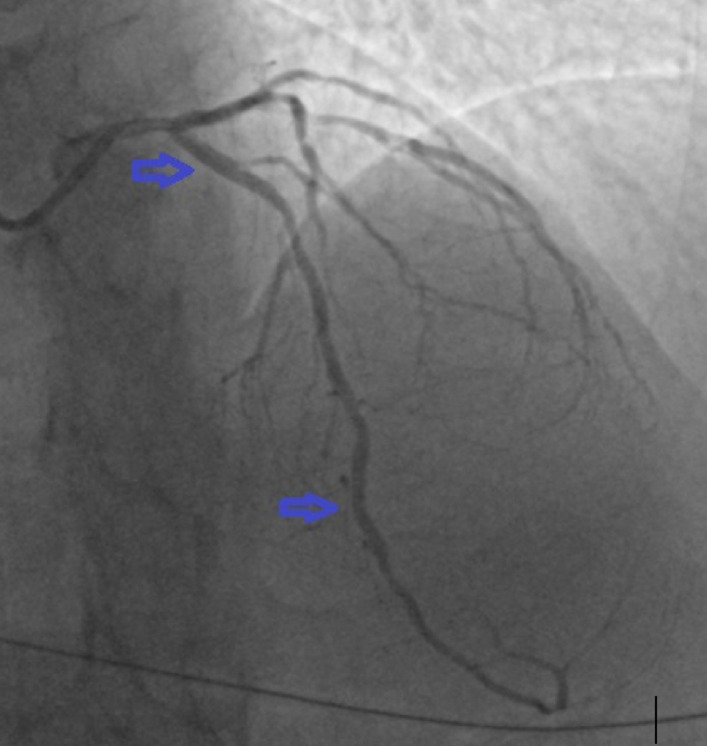
coronary angiogram: postero-anterior cranial showing the left anterior descending artery after stenting the proximal and medial lesions

**Figure 4:**
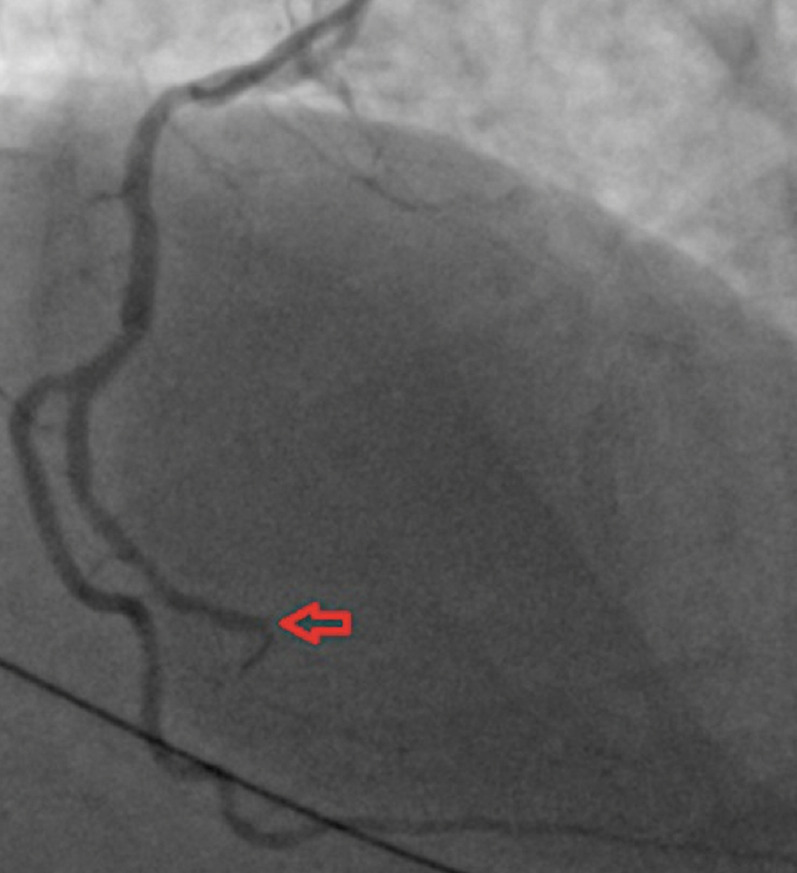
coronary angiogram: left anterior oblique cranial view showing chronic total occlusion of the mid right coronary artery (red arrow)

**Figure 5:**
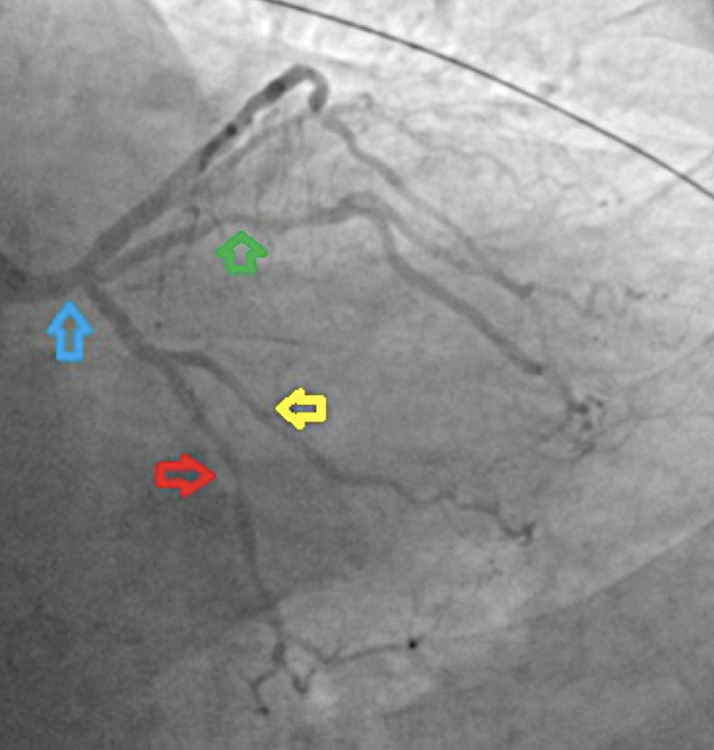
coronary angiogram: left anterior oblique caudal view showing 50% stenosis (blue arrow) of the distal left main stem, 80% proximal stenosis of the ramus (green arrow), 60% stenosis of the mid left circumflex artery (red arrow), and 50% stenosis of the obtuse marginal 1 artery (yellow arrow)

**Diagnostic assessment:** coronary angiography showed multi-vessel disease involving the 80% stenosis of the left anterior descending (LAD) just before the first diagonal branch-likely culprit, a 50% stenosis of the left circumflex (LCX) and 100% stenosis of right coronary (RCA-likely CTO) as seen in Figure 2, Figure 3, Figure 4, and Figure 5.

**Therapeutic intervention:** a 2.5*18 mm drug-eluting stent (DES) was deployed in mid-LAD, and another 2.75*12 mm DES was deployed in the proximal LAD, as seen in Figure 3. Admitting troponin I levels were found to be at 25,000 ng/ml (0-17). Further laboratory workup revealed a deranged lipid profile with elevation in total cholesterol (6.8 mmol/l) and LDL (5.19 mmol/l). A transthoracic echocardiogram (TTE) performed one hour post-PCI showed left ventricular ejection fraction (LVEF) of 40–45% with apical akinesia. His admission was uneventful, and he was subsequently discharged after 72 hours on the following medications: aspirin 75 mg once daily (OD), prasugrel 10 mg OD, atorvastatin 80 mg OD, bisoprolol 5 mg OD, eplerenone 12.5 mg OD, dapagliflozin 10 mg OD, and metformin/vildagliptin 500/50 mg OD.

**Timeline:** despite pharmacological compliance, the patient was brought back to our hospital 72 hours post-discharge owing to increased dyspnea and worsening chest pain. On examination, the patient was in respiratory distress, having cold and clammy extremities. Initial vitals were BP of 88/50 mmHg, PR: 110 beats/min, RR of 28 cycles/min. Thoracic auscultation revealed rales bilaterally in the lower third of both lung fields. ECG anterior ST-segment elevation, similar to the recent admission ECG. The patient was started on non-invasive ventilation (NIV) and received intravenous frusemide and nor-epinephrine to relieve congestion and improve perfusion.

**Figure 6:**
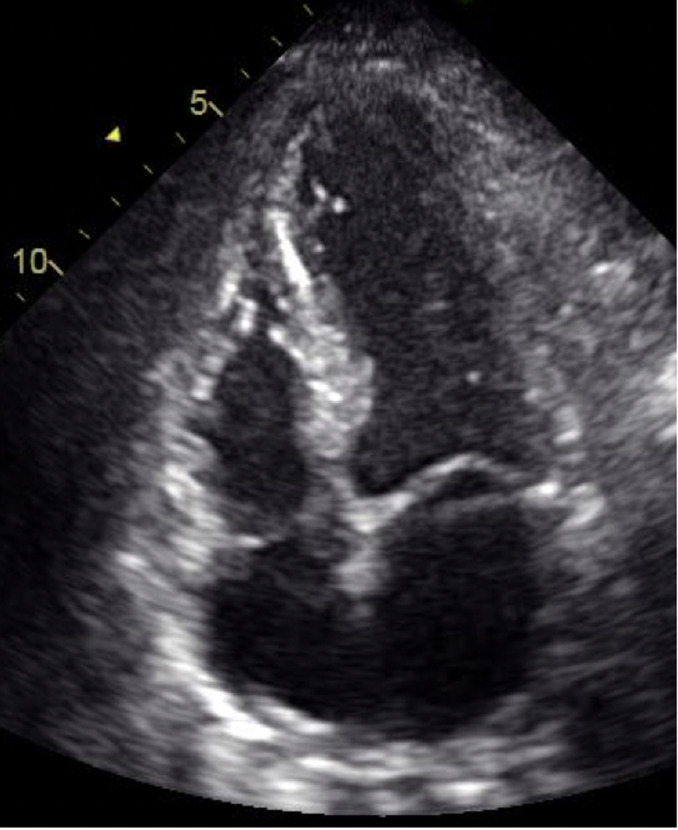
end-systolic frame of apical 4-chamber demonstrating features of apical ballooning suggestive of Takotsubo cardiomyopathy

**Figure 7:**
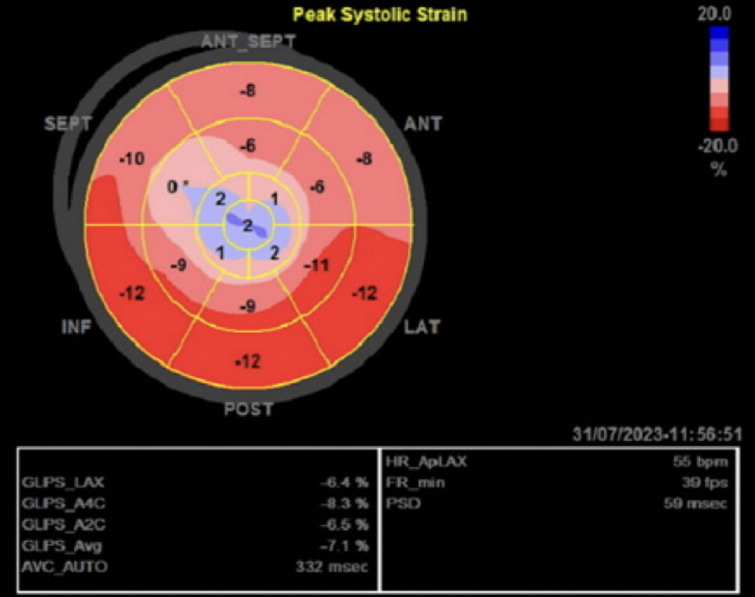
global longitudinal strain imaging showing areas of strain sequence

**Follow-up and outcomes:** a relook coronary angiography showed patency of the LAD stent. There were no new lesions. A few hours later, the patient exhibited significant clinical and hemodynamic improvement. A repeat TTE revealed a drop in LVEF to 25% with apical ballooning as seen in Figure 6 and Figure 7. No LV thrombus was identified. A cardiac magnetic resonance imaging (MRI) could not be performed because of claustrophobia. The patient was discharged on day 4 post-readmission. During review in clinic after two weeks, a follow-up TTE done as an outpatient revealed an improved ejection fraction of 45-50% without apical ballooning. The chronic total occlusion (CTO) to the RCA was treated electively after 5 months with good results, and the lesions in the circumflex were deemed appropriate for medical therapy. The patient is currently asymptomatic.

**Patient perspective:** the patient stated immense relief upon learning that the repeat coronary angiography showed no signs of stent thrombosis. He described the symptomatology, fearing the worst. He felt a significant burden lifted off his shoulders. The patient conveyed his deep gratitude to the medical team, acknowledging the swift and thorough care that contributed to this positive outcome.

**Patient consent:** individual verbal consent was obtained prior to writing the case report without raising concerns or reservations. The case report doesn´t include any patient identifier, and the patient´s confidentiality was maintained.

## Discussion

Takotsubo cardiomyopathy (TCM) is an acute reversible condition that clinically mimics AMI and occurs in the setting of physical or emotional stress in the preceding 1-5 days. The current prevalence of TCM among patients undergoing coronary angiography for suspected ACS is between 1% and 3% [1,2]. Normal coronary arteries are part of the major diagnostic criteria [3] and are regularly detected in TCM. Nonetheless, several CAD cases have been reported [1-3]. Registry findings from North America and Japan reported concomitant CAD in approximately 10%-40% of TCM [1,2]. TCM is more common among post-menopausal women and in older adults with a mean age between 60 and 76 years [1,2].

The incidence of post-MI TCM, however, is not well-known and likely under-documented. Suggested mechanisms are mostly due to catecholamine excess state [1] that exists during and after the ACS, compounded by the emotional stress [1,2] and fear the patient experiences. Additionally, multivessel spasm [1-3] and microvascular dysfunction [1-3] have also been hypothesized. Our patient had significant concern during his index hospital admission about financing his care, which meant he couldn´t receive non-culprit revascularization, and it was possibly an added factor to the development of TCM. TCM post-MI poses a diagnostic challenge since mechanical complications of acute MI and stent thrombosis present in a similar fashion [4]. 2D echocardiography is the most useful tool [4,5], especially in resource-limited settings, in differentiating the two in emergent situations [1]. Additionally, few studies have reviewed the value of ECG in discriminating TCM and anterior MI [1,2]. Interestingly, lack of ST-segment elevation in V1 and presence of ST-segment elevation in augmented vector right (aVR) with reciprocal changes has more than 90% sensitivity and specificity for TCM [1]. To date, no laboratory investigations, values, or trends have reported the ability to differentiate between TCM and ACS [2]; hence, further research in this field is warranted. Coronary angiography remains the only intervention in eliminating obstructive coronary disease in the list of differentials [4,5], as seen in our patient. Recently, there have been studies suggesting that gadolinium-enhanced cardiac MRI has the ability to distinguish Takotsubo cardiomyopathy from acute MI and myocarditis [6], an investigation not readily available in many sub-Saharan African centers.

Despite TCM being a reversible condition [1,2], hemodynamic and electrical instability can occur in one-fifth of patients, leading to serious in-hospital adverse events [1,2]. The in-hospital rates of adverse outcomes that include malignant arrhythmia, cardiogenic shock, LV thrombus, free wall rupture, and ventricular septal rupture are all comparable between the two disease entities [6,7]. Factors preceding the aforementioned complications include male sex [8] and a physical trigger [8], with a few case reports suggesting a 10-fold additional risk in patients with elevated troponin [9], as seen in our patient. It is also important to highlight that recurrent rates for TCM are variable and can recur even within a week [9]. Unfortunately, to date, there have been no documented factors to predict the likelihood of recurrence [9]. Fortunately, we were able to discharge our patient alive on goal-directed medical therapy for heart failure and appropriate secondary prevention for ACS. Nevertheless, patients with TCM have an in-hospital rate of all-cause mortality between 6-8% [10].

## Conclusion

This case documents an under-described and under-recognized phenomenon - the development of acute TCM induced by the stress of an AMI. It is essential for health care workers to vigilantly evaluate patients presenting with symptoms suggesting either condition. This condition, in most cases, resolves with continuation of medical therapy. To our knowledge, this is the first such report from the African continent.
